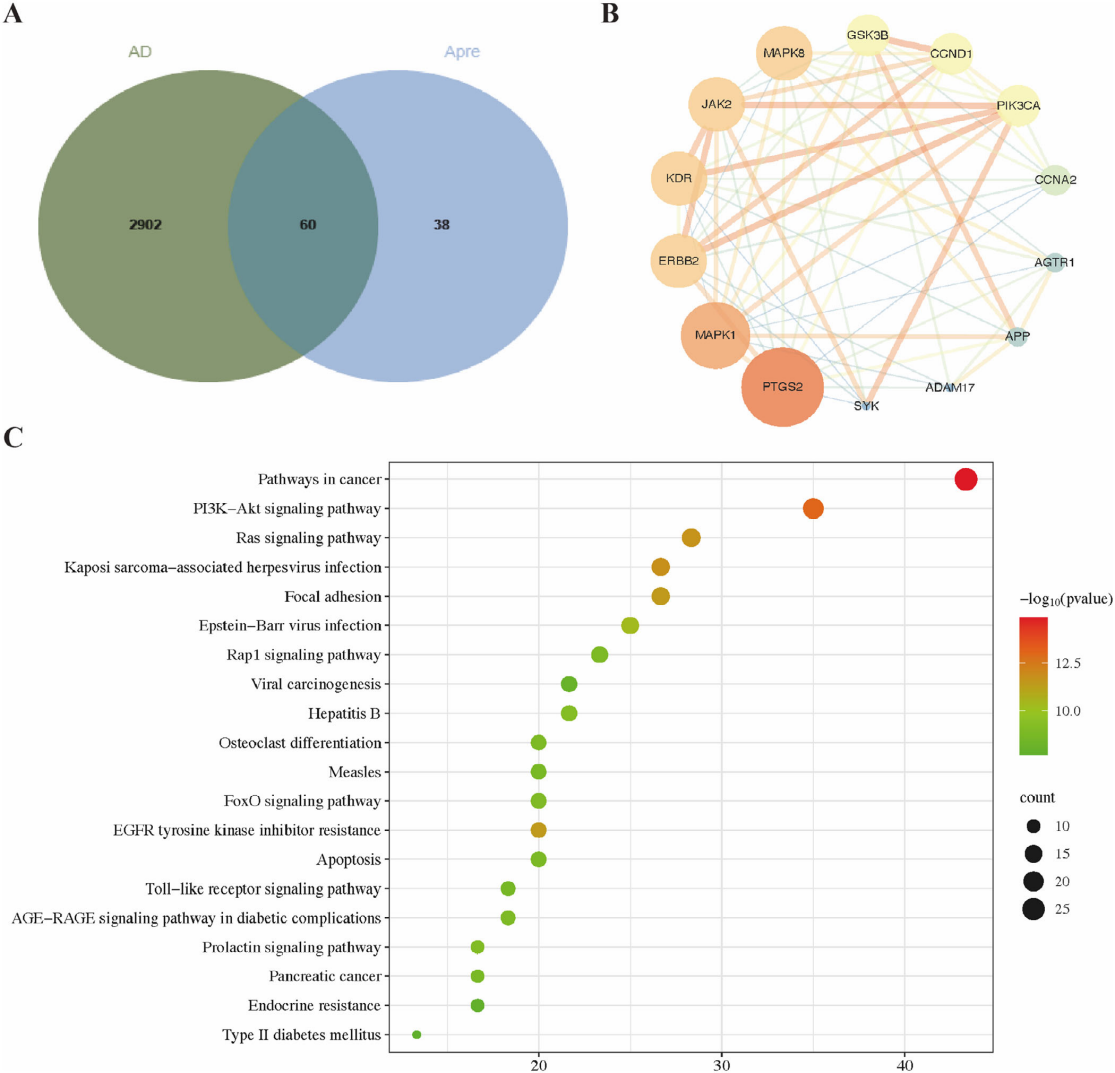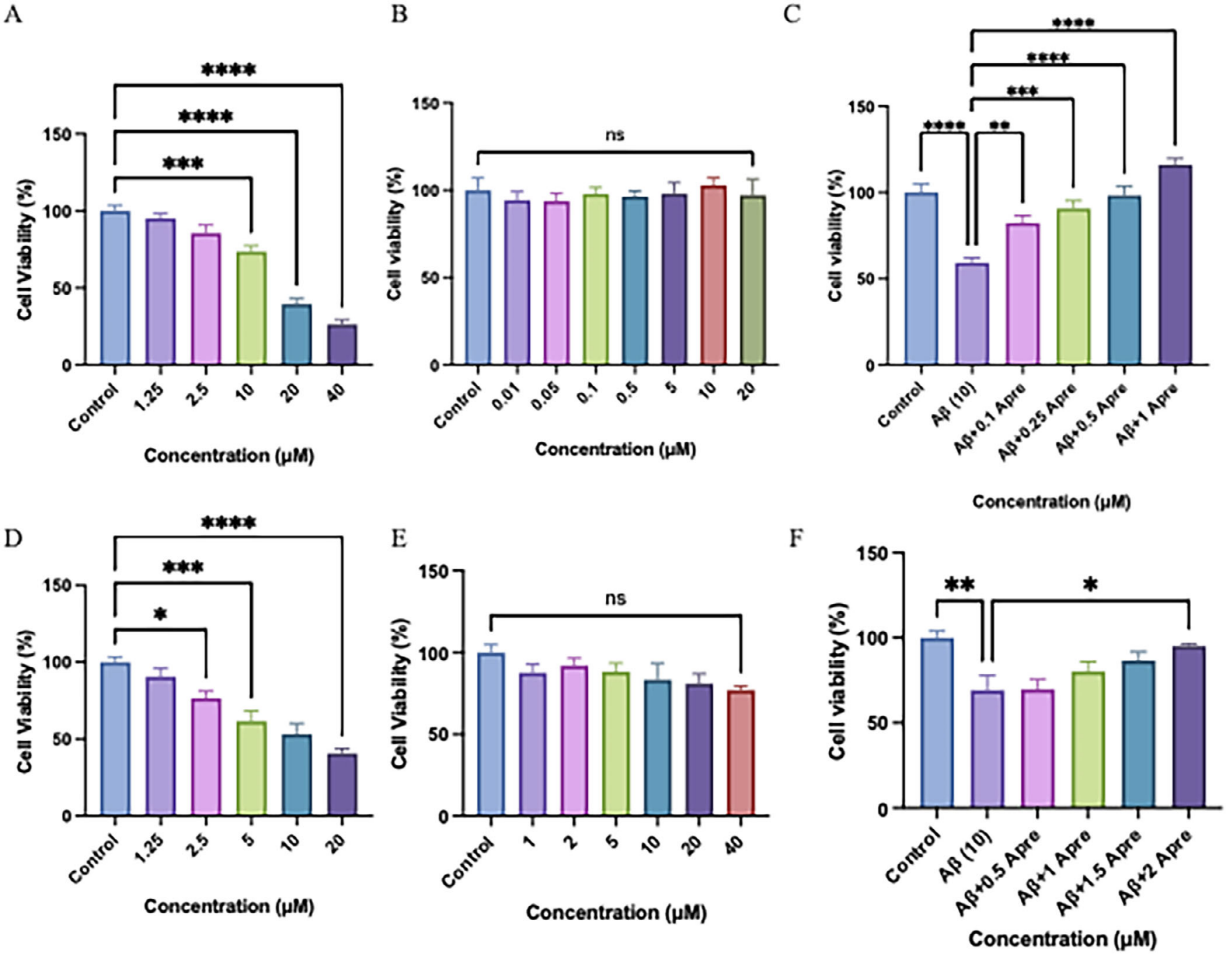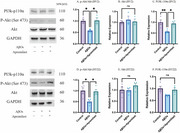# Apremilast Alleviates Aβ Oligomer‐Induced Cytotoxicity via the PI3K/Akt Pathway

**DOI:** 10.1002/alz70859_102959

**Published:** 2025-12-25

**Authors:** Hao Yang

**Affiliations:** ^1^ Xuanwu hospital Capital Medical University, Beijing, Beijing China

## Abstract

**Background:**

Apremilast, a novel inhibitor of phosphodiesterase‐4 (PDE4), has demonstrated anti‐inflammatory, immunomodulatory, neuroprotective, and senolytic properties. Given these characteristics, Apremilast, similar to other PDE4 inhibitors, holds potential as a therapeutic candidate for Alzheimer’s disease (AD). This study aims to investigate whether Apremilast can mitigate neurotoxicity induced by amyloid β oligomers (AβOs) in BV2 microglial and HT‐22 hippocampal mouse cell lines, while also exploring its neuroprotective effects and the underlying molecular mechanisms.

**Method:**

To begin, network pharmacology was employed to identify potential shared targets between Apremilast and AD. Molecular docking was subsequently used to assess the binding affinity of Apremilast to key targets. At the cellular level, the Cell Counting Kit‐8 (CCK‐8) assay was conducted to evaluate Apremilast’s protective effects against Aβ oligomer‐induced cytotoxicity in BV2 and HT‐22 cells. Finally, Western blot (WB) analysis was performed to examine the expression of proteins in the PI3K/Akt signaling pathway, offering insights into the molecular mechanisms underlying Apremilast’s neuroprotective role.

**Result:**

Target enrichment analysis identified several potential pathways, among which the PI3K/Akt pathway was chosen for further examination. The results showed that Apremilast effectively counteracted Aβ oligomer‐induced cytotoxicity in both BV2 and HT‐22 cells, leading to a significant improvement in cell viability. Moreover, Western blot analysis demonstrated an upregulation of phosphorylated Akt (p‐Akt/Akt) and PI3K protein levels, suggesting that activation of the PI3K/Akt pathway may play a crucial role in the neuroprotective action of Apremilast.

**Conclusion:**

In conclusion, this study provides evidence that Apremilast can protect against AβOs‐induced cytotoxicity in BV2 and HT‐22 cells by modulating the PI3K/Akt signaling pathway, supporting its potential application in future clinical research.